# Prevalence, outcome and quality of care among children hospitalized with severe acute malnutrition in Kenyan hospitals: A multi-site observational study

**DOI:** 10.1371/journal.pone.0197607

**Published:** 2018-05-17

**Authors:** Susan Gachau, Grace Irimu, Philip Ayieko, Samuel Akech, Ambrose Agweyu, Mike English, on behalf of the Clinical Information Network Author Group

**Affiliations:** 1 Kenya Medical Research Institute -Wellcome Trust Research Programme, Nairobi, Kenya; 2 Department of Paediatrics and Child Health, University of Nairobi, Nairobi, Kenya; 3 Nuffield Department of Medicine, Oxford University, Oxford, United Kingdom; Institut de recherche pour le developpement, FRANCE

## Abstract

**Background:**

Severe acute malnutrition (SAM) remains a major cause of admission and inpatient mortality worldwide in children aged less than 5 years. In this study, we explored SAM prevalence and outcomes in children admitted in 13 Kenyan hospitals participating in a Clinical Information Network (CIN). We also describe their immediate in-patient management.

**Methods:**

We analyzed data for children aged 1–59 months collected retrospectively from medical records after discharge. Mean, median and ranges were used to summarize pooled and age-specific prevalence and mortality associated with SAM. Documentation of key signs and symptoms (S/S) and performance of indicators of quality of care for selected aspects of the WHO management steps were assessed. Logistic regression models were used to evaluate associations between documented S/S and mortality among SAM patients aged 6–59 months. Loess curves were used to explore performance change over time for indicators of selected SAM management steps.

**Results:**

5306/54140 (9.8%) children aged 1–59 months admitted with medical conditions in CIN hospitals between December 2013 and November 2016 had SAM. SAM prevalence identified by clinicians and case fatality varied widely across hospitals with median proportion (range) of 10.1% (4.6–18.2%) and 14.8% (6.0–28.6%) respectively. Seventeen variables were associated with increased mortality. Performance change over time of management steps varied across hospitals and across selected indicators but suggests some effect of regular audit and feedback.

**Conclusion:**

Identification of SAM patients, their mortality and adherence to in-patient management recommendations varied across hospitals. An important group of SAM patients are aged less than 6 months. Continued efforts are required to improve management of SAM in routine settings as part of efforts to reduce inpatient mortality.

## Introduction

Severe acute malnutrition (SAM) is a significant direct or indirect contributing factor in approximately half of the 5.9 million deaths of children aged under 5 years worldwide [1–4]. In 2013, World Health Organization (WHO) updated anthropometric diagnostic criteria to include mid-upper arm circumference (MUAC) as an alternative to weight/height Z-score in children aged 6–59 months [5]. The addition of this technologically simple diagnostic tool was expected to improve recognition of SAM. For those with SAM complicated by acute or severe illness, WHO recommends in-patient hospital care. To improve outcomes of SAM hospital care, WHO developed 10-management steps as part of clinical guidelines in 1999 that have been updated most recently in 2013 [5]. It was expected that adherence to these steps by health care providers would reduce mortality to less than 10% [5]. However, adherence to the treatment guidelines is a challenge and mortality rates of 10–40% among SAM hospitalized children have been reported in studies from sub-Saharan Africa, though these often represent single hospital settings [6, 7]. Understanding the quality and outcomes of complicated SAM in wider routine hospital settings is typically limited by lack of appropriate data for monitoring hospital care [8, 9].

In a broad effort to improve quality of care for the seriously sick child, the Kenya Ministry of Health in collaboration with partners including the KEMRI-Wellcome Trust Research Programme and the Kenya Paediatric Association developed Basic Paediatric Protocols that give guidance on the clinical management of common causes of inpatient morbidity and mortality[10, 11]. Protocols describe essential signs and symptoms in patients’ assessment, syndromic classification, essential laboratory investigations and choice of treatment and dosages [10, 12]. Linked to the protocols is a structured paediatric admission record (PAR) form and a 5-day training course (Emergency Triage Assessment and Treatment PLUS admission care, ETAT+) [10, 13] for their dissemination [14]. To monitor uptake of recommendations, the same partners have worked since 2013/2014 with 14 county hospitals to establish a Clinical Information Network (CIN). We have described the CIN in detail elsewhere [8, 11, 15, 16]. In brief, the aim of CIN is to work with hospitals to improve documentation practices and utilize data to promote guideline adoption through regular audit and feedback reports.

Here we describe a longitudinal observational study to determine the prevalence and outcomes of SAM in children aged 1–59 months admitted to the CIN hospitals. Additionally, we describe performance metrics for the in-patient care of complicated SAM for patients aged 6–59 months with regard to quality of documentation, identification of patients with SAM and their immediate in-patient management. We also explore risk factors associated with SAM mortality among children aged 6–59 months.

## Methods

### Study design and setting

This was a longitudinal hospital-based observational study. Management of children with SAM was monitored at hospital level over nearly three years as part of an on-going large multifaceted pragmatic study that aims at improving quality of documentation practices and utilization of data to improve care in county hospitals [8, 11, 16, 17]. Four of the hospitals joined the CIN in September 2013, six in October 2013 and four in February 2014. However, we chose a monitoring period of December 2013 to November 2016 for this study. Thirteen of the hospitals have moderate to high inpatient workload. One hospital that has a low in-patient workload and where only clinical officers (non-physician clinicians) provide care is not typical of most county referral hospitals and was therefore excluded from this analysis [8]. In each hospital, the hospital paediatrician, the nurse in-charge of the paediatric wards and the head health records information officer are the CIN focal persons and form the link between the collaborators and the hospital in delivering any intervention. Details of the process of engaging hospitals in this network intervention are described elsewhere [11, 18]. In brief, since its inception in September 2013, CIN has encouraged use of the PAR, a linked summary discharge form and the Basic Paediatric Protocols. Each hospital is given an audit report every 2–3 months demonstrating the documentation practices and health workers’ performance using the Basic Paediatric Protocols recommendations as the audit criteria. The dissemination of the audit report within hospitals is facilitated by the CIN focal persons under the leadership of the hospital paediatrician. The CIN focal persons are invited for a meeting once a year to build their leadership skills to introduce and sustain change in their respective hospitals [16]. The CIN paediatricians are invited for an additional meeting once per year to build their capacity to facilitate the audit feedback hospital meetings, interpret the performance indicators, facilitate problem solving and discuss the strategic plans for the CIN [11, 16, 18].

The county hospitals are administratively and financially supported by their respective county governments. The research team supports data collection, analysis and dissemination of audit reports only. No financial or material resources are provided for the management of SAM patients except for copies of protocol booklets, while mid-upper arm circumference (MUAC) tapes were provided to hospitals by UNICEF beginning in May 2014 after advocacy from CIN partners.

### Study participants

We examined the database of admission episodes and considered for the purpose of this study that all children aged 1–59 months admitted to the hospitals’ paediatric wards were deemed eligible if they could be allocated to one of three mutually exclusive groups. Allocation to one of these groups was based on analyses of available patient data and each individual case was assigned, in order, to group A, B or C depending on the criteria outlined below.

i) Group A—Explicit SAM diagnosis—the admitting clinician assigned an admission diagnosis of severe acute malnutrition, severe malnutrition, marasmus, marasmic-kwashiorkor or kwashiorkor
ii) Group B—Implicit SAM diagnosis—the admitting clinician prescribed either F75 (recommended initial feed for complicated SAM) and/or ReSoMal (recommended oral rehydration solution for SAM) without making an explicit diagnosis of SAM (so not meeting criteria for Group A)
iii) Group C—Retrospective SAM diagnosis- Children not meeting criteria for Group A or B were classified as belonging to this group if MUAC (or WHZ score) documented was consistent with SAM or there was presence of oedema not attributable to renal or cardiac causes.

Surgical and burn cases, and those with renal diseases, liver diseases, or heart diseases were excluded from analyses. For some periods, only a minimum dataset was collected for patients admitted during industrial strikes or data clerks’ annual leave. These minimal data are restricted to biomedical and outcome information that is required by the national reporting system. A similar minimum dataset was also collected in random samples of 60% to 30% cases from two very busy hospitals to ensure data clerks could manage the workload [15]. All children with only a minimum dataset were excluded from analyses.

### Data collection

Trained data clerks collect the data from routine case records for the paediatric ward admissions at discharge across the CIN hospitals as described elsewhere [8, 15, 19]. The case records consist of the PAR, a structured discharge summary, laboratory order and results forms, treatment charts, and fluid/feed intake monitoring charts. Data on patient demographic information, clinical assessment, diagnosis and classification of illness severity, laboratory and radiological investigations and patient outcomes are entered directly into a non-propriety Research Electronic Data Capture (REDCap) tool, with inbuilt range and validity checks. Data entry is guided by a standard operational manual that forms the basis of the training of the data clerks. Daily error checking is done on site and also after the data are synchronized to a central server using automated procedures that prompt cross-checking of possible errors with the source documents and correction of errors [15]. External data quality assurance is done by research assistants who visit each hospital every 3 months and re-enter data from 5% of randomly selected records to check consistency with the data clerks’ entries.

### Statistical analysis

We present pooled and age-specific (1–5 months and 6–59 months) analyses to examine the prevalence and mortality associated with SAM among children admitted in the CIN hospitals between December 2013 and November 2016. Summary statistics include frequencies, proportions, mean, medians and ranges for categorical and continuous variables as appropriate.

WHO and Kenyan protocols for management of complicated SAM are primarily aimed at children aged 6–59 months. Thus, among children aged 6–59 months, we assessed documentation of essential clinical signs and symptoms (S/S) used in classification of severity of illness of common serious illnesses[12]. This analysis was restricted to the period between April 2014 and November 2016 when all the 13 hospitals contributed data. We used the S/S to describe the characteristics of all SAM populations (stratified by group A, B and C). Those S/S with good levels of documentation (greater than 80%) were explored in univariable logistic regression models to evaluate their association with SAM mortality. Other patient characteristics considered in these analyses were; patient’s sex, age group, number of comorbidities, SAM group and malaria parasite slide status (positive or negative). The outcome of interest was a binary variable indicating patient’s outcome at discharge (dead or alive). Odds ratios (OR) and corresponding 95% confidence intervals were used to measure the magnitude and direction of association. Univariable models were based on complete cases analysis. Hospitals were treated as random effects (random intercepts) with the intra cluster correlation coefficient (ICC) used as a measure of variation between hospitals. In children aged 6–59 months in groups A and B, we used frequencies and proportions (mean, median and ranges) to describe performance of indicators of quality of care for selected aspects of the WHO and Kenyan 10 key management steps (Table 1). Loess curves with 95% confidence bands based on data from all the hospitals combined and where this was sufficient for the purpose were used to examine performance trends over time (April 2014 to November 2016) for management steps. All analyses were performed in R version 3.3.0. Alpha error was set at 0.05 for all statistical tests.

**Table 1:** Definition of quality of care indicators used to assess performance of clinicians in SAM management^1^.

Step	Indicator definition
Step 1	Proportion of SAM cases with random blood glucose (RBG) <3mmol/l or clinically possible hypoglycaemia who were prescribed 10% dextrose on the admission day^2^.
Step 2	Proportion of SAM cases who had axillary temperature recorded in the clinicians’ notes on the admission day
Step 3	A:—Proportion of SAM cases with diarrhea who had ReSoMal prescribed excluding those who had bloody diarrhea, were vomiting everything, had AVPU = Unresponsive or had a diagnosis of shock^3^.
	B:—Proportion of SAM cases with admission diagnosis of anemia or pallor and HB<4g/dL, or signs of severe pallor or Hb 4-5g/dl with respiratory distress who had blood transfusion ordered^4^.
Step 5	Proportion of SAM admissions, excluding readmissions, who had antibiotics (penicillin and gentamicin or ceftriaxone) prescribed on the admission day.
Step 7	A:—Proportion of SAM cases who had F75 prescribed during the period of admission.
	B:—Proportion of SAM cases who had correct daily volume of F75 prescribed. • 100ml/kg/24hrs (+/- 20%) for those with severe oedema. • 130mls/kg/24hrs (+/-20%) no oedema or if oedema present not severe.

^1-^Quality of care indicators are derived from Basic Paediatric Protocols and ETAT+ recommendations

^2^Clinically suspected hypoglycaemia- patients unable to drink or not alert on AVPU scale

^3^Shock- based on documented clinical signs -AVPU less than alert and absent/weak pulse and capillary refill > 3 seconds and temperature gradient

^4^Respiratory distress–Patients with grunting or lower chest indrawing or acidotic breathing

### Ethics approval

The Kenya Ministry of Health and Kenya Medical Research Institute’s Scientific and Ethical Review Unit approved the use of de-identified patient data obtained through retrospective review of medical records without individual patient consent.

## Results

**Fig 1:**
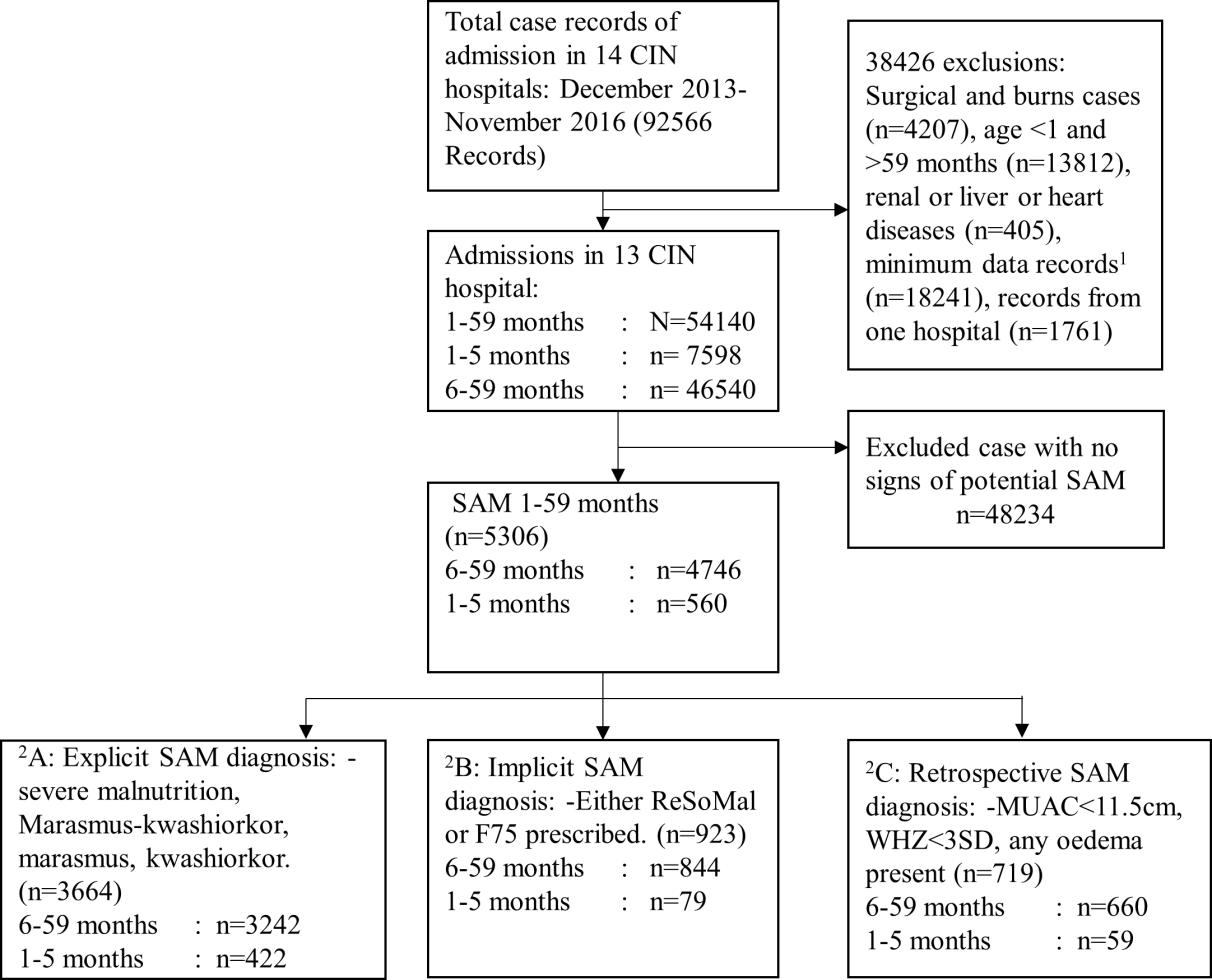
Flowchart of selection and classification of potential severe acute malnutrition cases in the CIN hospitals. CIN- Clinical Information Network, SAM: -Severe acute malnutrition, ^1^Minimum data records–data entry restricted to biomedical and outcome data that is required by the national reporting system ^2^Groups A, B and C are mutually exclusive.

The CIN routine health information database comprised of 92566 patients who were admitted between December 2013 and November 2016. Of these we excluded 41.5% (38426/92566) patients with age < 1 month, surgical conditions, burns and renal disease, liver disease, heart disease and those with minimum data or those admitted to one ineligible hospital. Of the 58.4% (N = 54140) case records that were analysed (Fig 1), 9.8% (n = 5306) had severe acute malnutrition (SAM) (groups A, B and C), their median admission age was 13 months with an interquartile range (IQR) of 8–20 months. Of 99.5% (5277/5306) children for whom sex was documented, 50.9% were males. The overall hospital mortality in the reference population was 5.9% (3217/54140) and SAM contributed to or was associated with 24.3% (781/3217) of these deaths. And among 48834/54140 (90.2%) patients with none of SAM subgroups A, B or C, mortality was 4.9% (2436/48834).

### Severe acute malnutrition prevalence and mortality among children aged 1–59 months admitted in the CIN hospitals

The median hospital prevalence of SAM among children aged 1–59 months, aged 6–59 months and aged 1–5 months, was 10.1% (5306/54140, range 4.6–18.2%), 10.4% (4746/46540, 4.8–20.4%) and 6.3% (560/7598, 2.6–15.9%) respectively with substantial variation across hospitals. Overall mortality among all SAM admissions aged 1–59 months was 15.8% (781/5306). Mortality rates varied substantially across hospitals with a median mortality of 14.8% (781/5306, 6.0–28.6%) and was higher among children aged 1–5 months with a median proportion of 17.9% (95 /560, 3.7–37.5%, Table 2) who accounted for 12% of all SAM deaths (95/781). By 72 hours of admission 60% (484/781) of all SAM deaths had occurred; with 53.1% (415) and 311 (39.8%) within 48 hours and on the day of admission respectively.

**Table 2:** Prevalence and mortality according to age among children admitted with severe acute malnutrition in clinical information network hospitals.

	SAM prevalence			SAM mortality		
Age group	n/N, %	Median %	Range %	n/N, (%)	Median (%)	Range (%)
**1–59 months (all groups A, B & C)**	5306 /54140 9.9	10.1	(4.6,18.2)	781 /5306 15.8	14.8	(6.0,28.6)
**1–5 months (all groups A, B & C)**	560 /7598 6.9	6.3	(2.6,15.9)	95 /560 19.5	17.9	(3.7,37.5)
6–**59 months (all groups A, B & C)**	4746 /46540 10.5	10.4	(4.8,20.4)	686 /4746 15.6	15.9	(5.7,28.4)
Group A: ^1^Explicit SAM diagnosis	3242 /4746 68.0	67.7	50.9,83.6	532/3242 18.4	18.7	(6.3,36.6)
Group B: ^2^Implicit SAM diagnosis^2^	844/4746 17.2	17.1	(7.2,28.9)	94/844 13.0	10.9	(0.0,44.0)
Group C: ^3^Retrospective SAM diagnosis	660/4746 14.8	10.9	(3.7,35.4)	60/660 7.7	7.1	(0.0,12.5)

Abbreviations: SAM: -Severe acute malnutrition

^1^Explicit diagnosis:—admission diagnosis documented as severe malnutrition, Marasmus-kwashiorkor, marasmus or kwashiorkor in children aged 6-59months

^2^Implicit diagnosis:—no explicit diagnosis of SAM but patient aged 6-59months prescribed ReSoMal or F75

^3^Retrospective diagnosis: Neither explicit nor implicit diagnosis.

#### SAM prevalence and mortality among children aged 6–59 months

Of the 4746 SAM patients aged 6–59 months there was variation across hospitals in the proportions with an explicit admission diagnosis of SAM (group A), an implicit diagnosis (group B) and those identified based on recorded anthropometric criteria at admission (group C) with median prevalence of 67.7% (3242 /4746, range 50.9–83.5%), 17.1% (844/4746, range 7.2–28.9%) and 10.9% (660/4746, range 3.7–35.4%) respectively (Table 2). Group A patients had the highest mortality (median, 18.7%) with marked variation across hospitals (range 6.3–36.6%) and this group accounted for 77.5% (532/686) of all deaths among children aged 6–59 months across SAM subgroups (Table 2). Among SAM patients (group A, B and C) aged 6–59 months, 1082/4746 (22.8%) patients had oedema (feet, knee or face) with a mortality rate of 15.3% (166/916). Approximately, 34.5% (1639/4746) and 5.5% (406/4746) SAM patients had MUAC <11.5 CM and WHZ <-3SD with mortality rates of 16.71% (274/1639) and 14.03% (57/406) respectively. Five percent (248/4746) had MUAC<11.5 cm and WHZ<-3 SD as a composite criterion with a mortality rate of 17.3% (43/248). In the retrospective SAM diagnosis subgroup (group C), all the 660 (100) patients had presence of oedema (foot, knee or face). Fifty percent (330/660) of SAM subgroup C patients had MUAC documented however, none had a measurement below 11.5 cm. Similarly, out of 28.6% (189/660) SAM subgroup C patients with documented WHZ measurement none had WHZ<-3 SD.

#### Preferred anthropometric measurement for assessment of acute malnutrition

**Fig 2:**
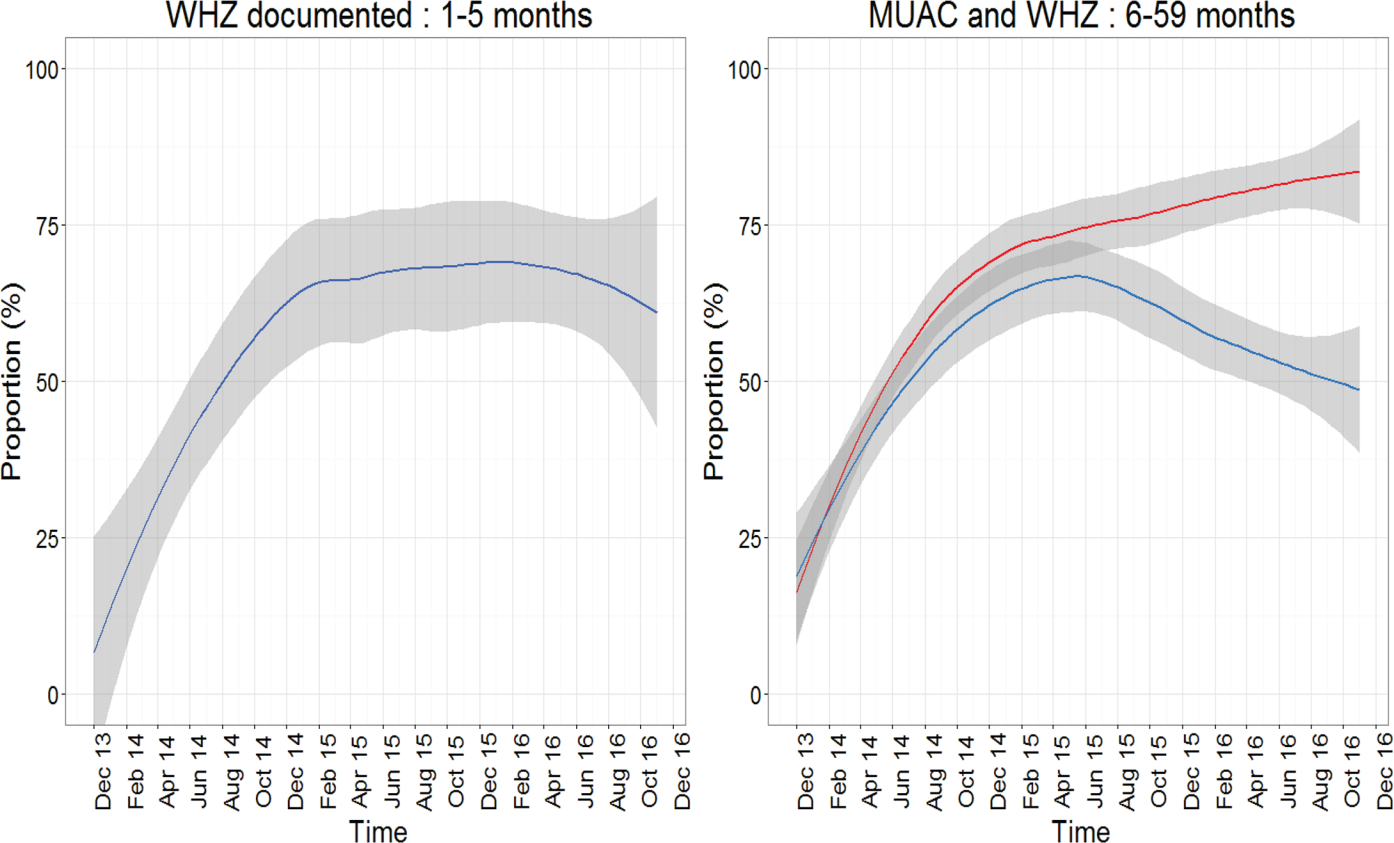
Documentation of WHZ score among all children 1 to 5 months (panel a) and WHZ score and MUAC documentation among all subgroups of SAM aged 6 to 59 months in 13 CIN hospitals over time (panel b): WHZ (blue line) and MUAC (red line).

Overall, 54.6% (29567/54140) case records had anthropometric measurement (WHZ score for 1–5 months and WHZ score/MUAC for 6–59 months) documented of whom 16.5% (4886/29567) had SAM. Screening of acute wasting using anthropometric measurements among all admitted patients aged 1–5 months and 6–59 months was documented for a median proportion (range) of 28.7% (1.0–57.6%) and 62.5% (23.2–85.5%) admissions respectively showing substantial variation across hospitals. Of the 560 infants aged 1–5 months and the 4746 children aged 6–59 months with SAM (across subgroups A, B and C), documentation of anthropometric measurements was higher than that of all hospitals admissions with median proportions of 56.1% (range 4.3–88.9%) and 78.9% (range 58.8–95.7%) respectively. In both age categories, there was a sharp increase observed in assessment using WHZ/MUAC after the initial months of CIN activities (Fig 2). This reached a plateau in 2015 and 2016 for those aged 1–5 months where WHZ remains the only screening measurement for acute wasting recommended by the Basic Paediatric Protocols. In children aged 6–59 months, Basic Paediatric Protocols recommends either MUAC or WHZ score for assessment of acute wasting. Though the MUAC tapes became widely available for use for inpatients in May 2014, it was not until a year later that there was a clear preference of use of MUAC with a co-incident fall in the documentation of WHZ score. (Fig 2, left panel).

### Documentation of clinical signs and symptoms and characteristics of patients (6–59 months) admitted with SAM (all groups A, B and C) between April 2014 and November 2016

Key clinical signs that would enable syndromic diagnosis of medical complications of SAM were documented in over 90% of the case records, except for temperature gradient and capillary refill time whose documentation was between 80–90% (Table 3). History of cough, vomiting and diarrhoea were the commonest presenting symptoms in 63% (2917/4633), 50.3% (2316/4610), and 46.3% (2132/4610) respectively in the study population. Thirteen percent (378/2539) of those with cough had cough for more than two weeks. Fifty percent (2189/4347) of the patients had axillary temperature above 37.5°C while 11% (522/4347) were hypothermic (axillary temperature below 36.5°C). Chest wall indrawing was present in a third (1516/4584) of the patients while a fifth (869/4448) of the patients were unable to drink. Twenty-four percent (1082/4481) had oedema out of whom 219/1082 (20.2%) had severe oedema. 0.9% (41/4469) children had signs consistent with shock as defined in the Basic Paediatric Protocols namely presence of temperature gradient, capillary refill time >3seconds, weak peripheral pulse and altered consciousness while 8.1% (373/4595) had severe palmar pallor. Of 79.9% (2585/3232) children for whom HIV status was documented between January 2015 and November 2016, 6.0% (156/2585) were HIV antibody positive. Nineteen percent of 963 children with an oxygen saturation documented between November 2015 and November 2016 had hypoxia, defined as oxygen saturation < = 89%. Central cyanosis was very rare at 0.9% (42/4633) (Table 3).

**Table 3:** Documentation of signs and symptoms important in the assessment and management of paediatric severe acute malnutrition among children aged 6–59 months: April 2014-November 2016.

Clinical sign/symptom during this illness	Number of records signs documented/N, (%) N = 4746	Patient characteristic	Presence of sign / Number of records signs documented, (%)
Axillary temperature	4347 (91.8)	Fever (>37.5°C)	2189/4347 (50.2)
		Hypothermia (<36.5°C)	522/4347 (11.9)
Cough	4633 (97.6)	Cough present	2917/4633 (62.9)
		Cough >2 weeks	378/2917 (12.9)
Diarrhoea	4610 (97.1)	Diarrhoea present	2132/4610 (46.3)
		Diarrhoea >2 weeks	160/2132 (7.5)
Vomiting	4604 (97.0)	History of vomiting	2316/4604 (50.3)
		Vomiting everything	1099/2316 (47.5)
Difficult feeding	4221 (95.3)	Difficult feeding present	1868/4221 (41.3)
Convulsions	4543 (95.7)	History of convulsions	459/4543 (10.1)
Oedema	4481 (94.4)	Oedema present (severe, mild/moderate)	1082/4481 (24.2)
Central cyanosis	4633 (97.6)	Central cyanosis present	42/4633 (0.9)
Chest indrawing	4584 (96.6)	Chest indrawing present	1516/4584 (33.1)
Grunting	4554 (96.9)	Grunting present	517/4554 (11.4)
Peripheral pulse	4364 (91.9)	Weak peripheral pulse	484/4364 (11.1)
Capillary refill time	4180 (88.1)	Capillary refill time >3 sec	96/4180 (2.3)
Palmar pallor	4595 (96.8)	Severe pallor	373/4595 (8.1)
Temperature gradient	3805 (80.2)	Skin warm up to elbow and shoulder	321/3805 (8.4)
Level of consciousness	4574 (96.4)	^1^Altered consciousness	339/4574 (7.4)
Ability to drink	4448 (93.7)	Unable to drink	980/4448 (22.0)
Stiff neck	4551 (95.9)	Stiff neck	121/4551 (2.7)
^2^HIV status known	2585/3232 (79.9)^2^	HIV antibody positive	156/2585 (6.0)
^3^Oxygen saturation	963/1721 (55.9)^3^	Hypoxia	187/963 (19.4)

^1^altered consciousness; verbal response = 93/339 (27.4%), Pain response = 192/339 (37.6), Unresponsive 54/339 (10.6%)

^2^HIV status known (positive antibody test): -January 2015-November 2016

^3^Oxygen saturation: -November 2015-November 2016. Hypoxia = Oxygen saturation (spO_2_) < = 89%

### Factors associated with mortality among children aged 6–59 months hospitalized with severe acute malnutrition

We evaluated association with SAM mortality for 21 socio-demographic and clinical factors in children aged 6–59 months admitted between April 2014 and November 2016. In univariable analyses 17/22 variables were significantly associated with SAM mortality while presence of cough, abnormal axillary temperature (below 35.6°C or above 37.5°C), vomiting everything, oedema of any level, and stiff neck were not (data not shown). Patients with implicit diagnosis (group B) and retrospective diagnosis of SAM (group C) had significantly lower risk of death compared to patients with explicit diagnosis of SAM (groups A) (OR 0.65;95% CI 0.51–0.84 and OR 0.42; 95% CI 0.31–0.56) respectively. The intra-cluster correlation coefficient (ICC range, 0.141–0.153) suggested moderate variation in outcome potentially associated with hospital level characteristics (Table 4).

**Table 4:** Complete cases univariable analysis for association with mortality among children admitted with severe acute malnutrition (all groups A, B and C) between April 2014 and November 2016.

Variable	OR (95%CI)	*P* value	ICC
^1^Age in months: 12–59	0.74 (0.62,0.89)	0.001	0.149
Child sex: Male	0.81 (0.68,0.96)	0.01	0.148
SAM: ^2^Group B ^3^Group C	0.65 (0.51,0.84)	<0.001	0.149
	0.42 (0.31,0.56)	<0.001	0.149
^4^Number of comorbidities: 1 2 > = 3	1.32 (0.96,1.8 4)	0.08	0.148
	1.84 (1.35,2.53)	<0.001	0.148
	2.16 (1.59,2.97)	<0.001	0.148
^5^Temperature: Fever (>37.5°C) Hypothermia (<36.5°C)	1.17 (0.97,1.43)	0.108	0.146
	1.24 (0.93,1.64)	0.136	0.146
Cough: Yes	1.06 (0.89,1.27)	0.521	0.148
History of diarrhea: Present	1.90 (1.60,2.27)	<0.001	0.147
Vomiting everything: Yes	1.13 (0.89,1.44)	0.299	0.141
Difficult feeding: Yes	1.31 (1.10,1.56)	0.003	0.149
Convulsions: Present	1.65 (1.27,2.13)	< 0.001	0.150
Oedema: Severe	0.78 (0.51,1.16)	0.239	0.149
Central cyanosis: Present	3.07 (1.54,5.84)	<0.001	0.147
Chest indrawing: Present	2.27 (1.88,2.74)	<0.001	0.153
Grunting: Present	2.54 (2.01, 3.2)	<0.001	0.148
Peripheral pulse: Weak	3.79 (3.02,4.74)	<0.001	0.146
Capillary refill time: >3 sec	3.25 (2.05,5.06)	<0.001	0.145
Palmar pallor: Severe	1.90 (1.45,2.47)	<0.001	0.145
Temperature gradient: warm up to elbow/shoulder	4.05 (3.09,5.29)	<0.001	0.148
Level of consciousness: VPU	7.31 (5.70,9.32)	<0.001	0.147
^6^Ability to drink: No	3.6 (2.9,4.3)	<0.001	0.150
Stiff neck: Yes	1.41(0.83,2.27)	0.178	0.148
^7^Malaria parasite slide: positive	0.63 (0.46,0.86)	<0.001	0.133

Abbreviations: ICC:—intra-cluster correlation coefficient; SAM:-severe acute malnutrition, AVPU: -Alert, Verbal response, Pain response, Unresponsive.

^1^Age in months reference group:- 6–11 months Reference; SAM reference group: Group A (Explicit SAM diagnosis).

^2^Group B—Implicit SAM diagnosis

^3^Group C-Retrospective SAM diagnosis

^4^Comorbidities:—Malaria, anaemia, tuberculosis, asthma, pneumonia, meningitis, HIV/AIDS, rickets, diarrhoea and dehydration.

^5^Temperature reference category: normal temperature between 36.5°C and 37.5^0^ C.

^6^Patients who are at AVPU = V, P, U are considered not able to drink if ability/inability to drink is not documented.

7Malaria parasite slide: positive- analysis restricted to 5 hospitals drawn from high endemic region in Kenya.

### Performance of clinicians in management of SAM cases aged 6–59 months

We evaluated performance of the clinicians in the management of 4086 patients aged 6–59 months who had either explicit or implicit SAM diagnosis (groups A and B) between December 2013 and November 2016 using our operational quality indicators as the audit criteria (Table 1). Table 5 depicts the performance of indicators across time and variation of performance across hospitals respectively. In general, performance varied across hospitals and management steps (indicators). Management of SAM cases for hypoglycaemia (step 1) was poor across hospitals with a median proportion of 17.3% (67/644) prescribed recommended 10% dextrose. In contrast, prescription of recommended antibiotics (penicillin and gentamicin or ceftriaxone) on admission day (step 5) and documentation of axillary temperature (step 2) at the point of admission had good performance across hospitals with median proportions (ranges) of 88.9% (2967/3345, range 66.9–97.3%) and 92% (3775/4086, 69.3–99.4%) respectively. Other management steps including prescription of ReSoMal for management of eligible diarrhoea cases (step 3A), blood transfusion in patients with severe pallor (step 3B) and prescription of F75 presented moderate performance, with medians of 31% (439/1266, range 3.0–56.7%), 41.7% (68/191, 6.3–77.8%) and 51.5% (2027/3892, 19.1–76.2%) respectively. Among SAM patients eligible for F75 in all the hospitals, only a quarter (946/3892, 10.6–38.0%) had their F75 prescribed in the right dose (volume and frequency in 24 hours adjusted for the severity of oedema). Performance of all indicators varied substantially across the CIN hospitals.

**Table 5:** Summary performance of clinicians in the management of SAM (group A&B) among children aged 6 to 59 months admitted to CIN hospitals^1^ between December 2013 and November 2016.

Management step	Proportion (%) of children eligible (pooled hospital data)	Proportion (%) with indicator achieved (pooled hospital data)	Median Hospital performance (%)	Range for hospital performance (%)
^2^Step 1: 10% dextrose prescribed	644/4086 (15.8)	67/644 (10.4)	17.3	(2.7,49.0)
Step 2: Axillary temperature	4086/4086 (100)	3775/4086 (92.4)	91.5	69.3,99.4)
Step 3 A: ReSoMal prescribed	1266/ 2093 (60.5)	439/1266 (34.7)	31.3	(3.0,56.7)
^3^Step 3 B: Blood transfusion	191/4086 (4.7)	68/191 (35.6)	41.7	(6.3,77.8)
Step 5 Antibiotics prescribed	3345/4086 (81.9)	2967/3345 (88.7)	88.9	(66.9,97.3)
Step 7 A: F75 prescribed	3892/4086 (95.3)	2027/3892 (52.1)	51.5	(19.1,76.2)
Step 7 B: Correct F75 dose	3892/4086 (95.3)	946/3892 (24.3)	23.5	(10.6,38.0)

^1^Analysis restricted to SAM subgroups A and B

^2^ Computation of median and range in management steps 1 restricted to 8 hospitals with at least 5 hypoglycaemia cases per hospital

^3^Computation of median and range in management steps 3B restricted to 11 hospitals with at least 5 severe pallor cases

### Trend of change in clinical assessment of SAM management indicators

**Fig 3:**
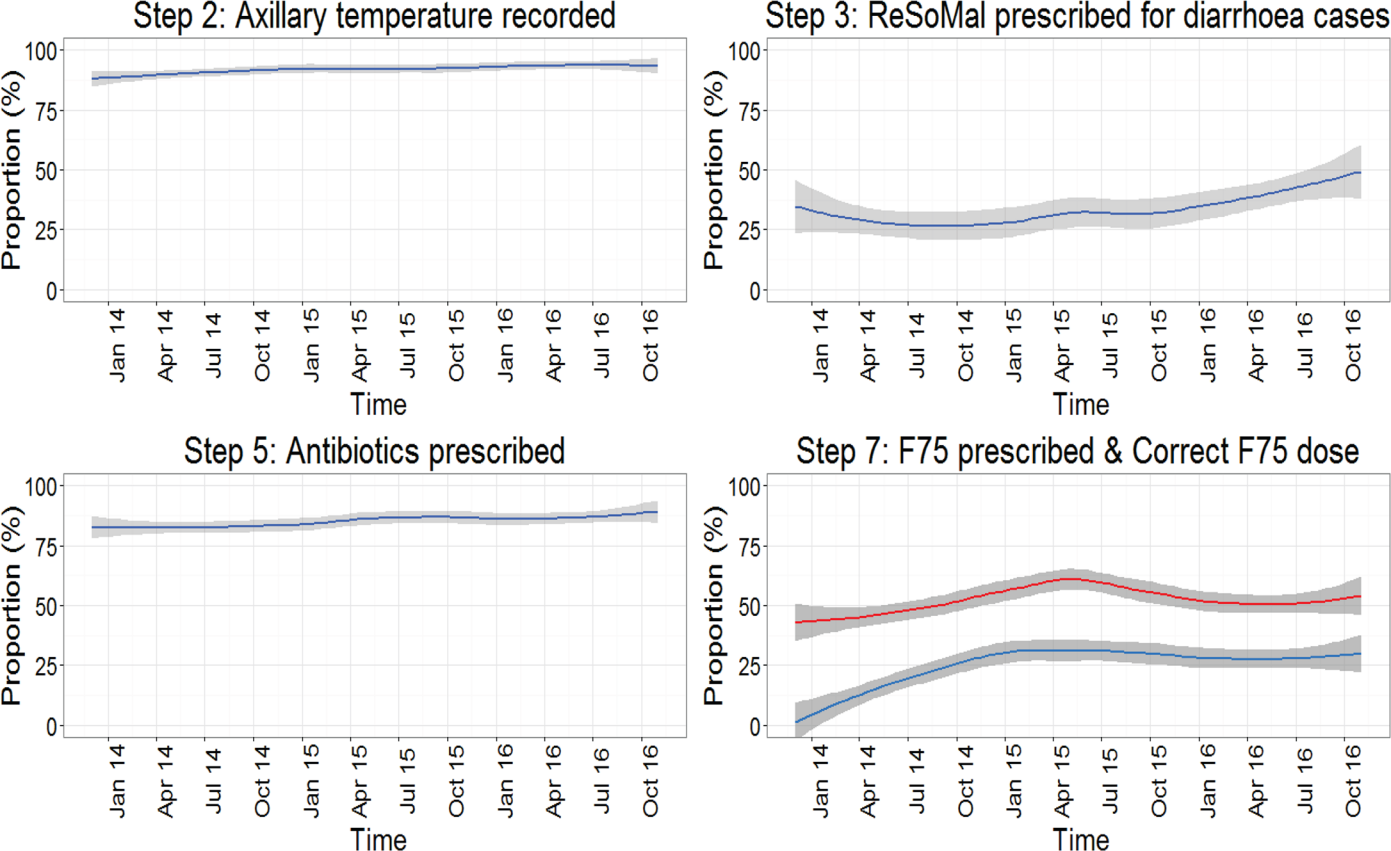
Cluster adjusted mean performance (solid line) with 95% confidence band portraying adherence to recommended guidelines over time for SAM management steps with sufficient data. Step 7: Red line is F75 prescribed and blue line is correct F75 dose.

We explored performance change over time for SAM management indicators with sufficient data and pooled across hospitals (Fig 3). Change in performance over time varied across indicators and the magnitude of change was dependent on baseline performance. In particular, documentation of axillary temperature (step 2) was high at baseline and exhibited minimal change over time (Fig 3, top left panel). Similarly, prescription of antibiotics (step 5) was moderately high at baseline, with a 9.1% performance increment between first and last follow up months (Fig 3, bottom left panel). Prescription of ReSoMal (step 3A) for those with diarrhea was evident in <50% cases throughout the monitoring period with a 23.6% performance change between first month and last month of study follow-up (Fig 3, top right panel). Prescription of F75 (step 7) to eligible SAM cases was moderate with a 21% performance increment between first month and last study period (Fig 3, bottom right panel, red line). Prescribing F75 in the right doses was a challenge in the first month of follow up, however, the accuracy of these prescriptions improved by 32% by the last month of the study period (Fig 3, bottom right panel, blue line).

## Discussion

Poor clinical documentation and weak data systems generally preclude meaningful analysis of adherence to guidelines in routine hospital settings [8, 9]. In this and other studies we have demonstrated that it is possible to establish a clinical information network in low and middle-income countries (LMICs) that provides valuable data to help understand routine care [8, 11, 20–22]. Despite childhood nutritional programmes targeting the under 5’s and recent good economic growth, severe acute malnutrition (SAM) remains a common admission diagnosis accounting for 10% of all medical admissions aged 1–59 months in 13 Kenyan county hospitals participating in the Clinical Information Network. In this study, prevalence of admission diagnosis of SAM varied widely across the hospitals. Although SAM was more prevalent among children aged 6–59 months, 6.3% of all infants aged 1–5 months had SAM (group A, B or C). Possible contributing factors to SAM in this age group may be low rates of exclusive breastfeeding and incorrect complementary feeding practices in Kenya [23]. Of the three SAM groups that we defined among patients aged 6–59 months, an explicit SAM diagnosis was the most common and had the highest mortality rate. Approximately 17% of SAM patients aged 6 to 59 months were identified retrospectively through anthropometric measurements consistent with SAM (group C). Although mortality was lower in SAM subgroup C compared to children with explicit and implicit SAM admission diagnoses (groups A and B), these patients missed out on recommended SAM care and are therefore at risk of progressing to more severe forms of SAM. Furthermore, none of the patients in SAM subgroup C had MUAC<11.5 CM or WHZ<-3SD. These results suggest that clinicians probably rely on the sign ‘visible wasting’ to identify SAM and appear to have a tendency of ignoring oedema as a sign of SAM.

In spite of Kenya endorsing and adopting the WHO guidelines for management of SAM [5, 12], we observed a median SAM mortality rate of 14.8% among children aged 1–59 months, higher than the WHO target of less than 10% [6]. Further, our data revealed a wide variation in mortality across the CIN hospitals and worse mortality in the infants aged 1-5months. In this study, SAM mortality rate is higher than the 5.7% and 7% reported earlier in Colombia and Hadiya in southern Ethiopia [24, 25] but lower than 21.3%, 38%, and 46% previously reported in southern Ethiopia, Kenya and Zambia respectively [26–28]. The variations in SAM mortality rate could be due to the differences in the causes of acute malnutrition in various parts of the world [29].

In the univariable analysis, clinical S/S including altered consciousness, weak pulse, inability to drink, temperature gradient, chest indrawing, diarrhoea and severe pallor were associated with increased mortality among SAM patients. Altered consciousness and severe palmar pallor were associated with mortality that could arguably be ameliorated by better adherence to step 1 and 3B. However, oedema, regardless of the severity, was not associated with increased SAM mortality contrary to findings elsewhere [30]. Additionally, hypothermia and hypothermia (abnormal body temperature) were not associated with SAM mortality. This lack of association may be attributed to poor clinical practices in documenting temperatures in routine settings [31]. A positive malaria slide was associated with lower SAM associated mortality in the 5 out of 13 CIN hospitals located in high malaria zones in Kenya [8]. A possible explanation is that undernourishment limits malaria parasite growth preventing SAM patients from progressing to severe malaria resulting in lower mortality as previously reported by Kakkilaya [32]. However, when severe illness does occur (e,g. severe malaria), malnourished children have a higher morbidity and mortality [32]. Furthermore, malaria as a co-morbidity is also probably readily treatable in SAM and may prompt admission with good outcome whereas other co-morbidities such as TB or bacterial infections may be less treatable.

The variation in mortality seen across hospitals in this study is likely explained in part by variation in the prevalence of these risk factors in the populations admitted. There was also some evidence (based on the ICC) that variation in mortality may be associated with the CIN hospital identity, a characteristic itself reflecting many possible influences spanning variability in the socio-economic status of catchment populations to the staffing ratios and many more [21].

The CIN strategy in the first two years of intervention was data driven feedback to enable hospitals to improve documentation practices [15]. Such audit and feedback is facilitated by having recognized audit criteria and developing staff capacity to act on audit findings [11]. Documentation of essential clinical signs and symptoms (S/S) rapidly improved and reached over 90% among SAM patients aged 6–59 months. Such documentation provides the basis for better characterizing patient groups and enabled the analysis of mortality risk factors. Cough, history of vomiting and diarrhoea were the most common symptoms while high body temperature, chest wall indrawing, oedema, inability to drink and hypoxia were the most common signs at presentation. Oedema occurrence was lower than in other studies [30] and we attribute this to the criteria used in the identification of SAM patients. In this study, MUAC was the preferred anthropometric measurement over time among 6–59 months old patients while documentation of WHZ score in infants less than six months improved over time. The rapid increase in documentation of MUAC was facilitated by improving the supply of MUAC tapes to the hospitals with CIN data used to advocate for their provision and use in line with current WHO severe acute malnutrition guidelines. Besides, MUAC is easier to measure and a better predictor of mortality than WHZ score [1]

Children with SAM have precarious physiology, and therefore require complex management that forms the basis of the globally recommended 10 management steps. Except for temperature assessment that had excellent performance at baseline, the selected processes of care indicators in this study all showed at least some improvement over time. However, the magnitude of change varied across indicators. Performance also varied substantially across hospitals despite all hospitals in the network receiving support (e.g. network participation and audit reports on prescription of ReSoMal and F75) [16]. Our results suggest that task complexity may influence both performance and performance change over time [33, 34]. Across hospitals, indicators that do not require a lot of cognitive effort on the part of clinician (e.g. documentation of body temperature by admitting clinician (step 2)) or representing existing routines of care (e.g. prescription of antibiotics either penicillin and gentamicin or ceftriaxone (step 5)) exhibited good performance during the entire study period. On the other hand, steps that were very specific to management of SAM [5] and which require greater cognitive effort such as prescribing F75 in the right doses (even though a reference table is provided in the Kenyan protocols) exhibited poor performance over the entire study period. The limited adoption of recommended practices in routine settings, although somewhat improved in the CIN, may also help explain why there has been little reduction in SAM (and SAM associated) mortality in some settings [9].

### Strengths and limitations

Our study involved a large number of cases over a sufficiently long study period enabling reporting of reasonably precise estimates. To our knowledge, this is the first audit report in an African setting to assess SAM prevalence and outcomes as well as quality of in-patient SAM care in multiple centers. A limitation of this study is that we rely on documentation of clinical features made by health workers in routine settings and are unable to determine if patients received prescribed care which is a problem in other settings [9]. Additionally, we do not have data on the availability of essential commodities that support the delivery of basic quality care for SAM patients [5, 12]. Our categorization of SAM patients was also retrospective and based on available data. For these reasons, our estimates of prevalence and mortality may not be directly comparable to those in studies employing careful, prospective surveillance. Furthermore, our routine data lacked socio-demographic and economic information of the study participants limiting our ability to assess important associations with SAM.

We also had to define indicators for SAM management steps, 1, 2, 3, 5 and 7 based on the data available and these may differ from those reported elsewhere [30]. Improving documentation practices of health workers much more widely would potentially allow comparative performance assessment across different settings and over longer periods.

### Conclusion

This study has demonstrated that SAM prevalence and mortality varies across hospitals and in some of the hospitals inpatient mortality is three time higher than the WHO target of less than 10% [6]. For children aged 6–59 months seventeen factors such as altered consciousness and severe palmar pallor were associated with increased SAM mortality. SAM is also an important diagnosis in infants less than 6 months who are at higher risk of SAM associated mortality. Adherence to indicators representing elements of the 10 steps management approach suggest adoption of key recommended practices such as appropriate feeding is a persistent challenge although there is some suggestion that continued audit and feedback associated with professional support and interaction in the form of network participation can help improve practices.

## Supporting information

S1 File (ZIP)
